# How Shall We Practise Dentistry in Order to Make It Healthful?

**Published:** 1891-12

**Authors:** Charles T. Terry

**Affiliations:** Milan, Italy


### HOW SHALL WE PRACTISE DENTISTRY IN ORDER TO MAKE IT HEALTHFUL?[1]

BY DR. CHARLES T. TERRY, MILAN, ITALY.

How often we hear it said that our calling is not a healthful one, and how often we think so ourselves; but, is it really the fault of our occupation?

---

[1] Read before the American Dental Society of Europe, at Heidelberg, August 4, 1891.

This is a question which interests us all. The influence of strong muscles, steady nerves, a delicate touch, and a level head on the salvation of teeth speak for themselves, and that these are merciful possessions in a dentist every patient whom we treat will testify, from the comfort and confidence which they give.

My experience of thirty-two years in our profession has convinced me that of all the different materials which have been used for filling teeth, gold, and gold combined with other materials, in the hands of careful, skilful dentists with good judgment, stands far in advance of everything else.

What material is there at the present day which enables the enterprising, skilful, conscientious dentist to accomplish what he knows to be his duty to his patients that can take the place of this material?

With gold, generally speaking, you are master of the situation in strengthening frail walls, enclosing exposed dentine, contouring, etc. Not only this, but with what other material can we work and leave the indelible impression on the minds of our patients that we are systematic, scientific, and skilful workers?

Using gold as often as it ought to be used, in order to obtain the best results in preserving decayed teeth and preventing decay in other teeth, requires an educated judgment, educated muscles, educated nerves, and very hard work, and compels the operator to be systematic, thorough, and painstaking, and to devote a certain amount of time undisturbed to each patient. The hard work which a first-class gold operation requires of the dentist who does not allow his patients to hurry him is beneficial to health, as all hard work is, provided it is not carried to excess.

Filling teeth with other materials does not require the same physical effort as with gold, and this physical effort, in my opinion, counteracts to a great extent the effects of confinement to, and bad air in, the operating-room.

Our best operators in the United States are healthy men, and they bear their age well. The dentist who is interested in his profession finds enough that is intellectual in his calling to keep his mind occupied and healthy. He comes in contact also with refined people, which is beneficial,—a change of patients giving relief and rest and a healthy variety to his work.

When our hours for receiving are over our cares end for the day, but with the physician it is quite different. His duties are irregular and connected with danger at times, and great responsibility such as we seldom encounter.

Our calling leaves us free to devote a certain part of each day to physical exercise and recreation, and I am convinced the dentist who pays attention to his physical condition can accomplish more as a delicate operator than one who goes on regardless of regularity and health.

A light breakfast and lunch of wholesome food, taken one hour before work, is important for health in our profession. After a hearty meal one ought not to work for several hours.

I have noticed that mountain-climbing is always accompanied with much more fatigue and exhaustion if undertaken after a hearty meal.

Dyspeptics will generally go on a canter immediately after eating heartily, thinking to get relief which they may imagine comes of physical effort, while actual digestion takes place only after they are exhausted and lie down to rest.

Our natures must be very tough to stand the many violations to the laws of health which we are constantly committing. Unfortunately, too many of us do not stand these violations.

The monkey can teach us how to eat. If we watch him, we notice how careful he is to reject the indigestible and irritating parts of everything he consumes, and how carefully he chews everything that enters his stomach.

After my experience in dentistry, I have become firmly convinced that our profession, as we can practise it abroad or at home, is one of the most favorable of all the professions for health and long life, provided we practise it with the intention of sustaining the high reputation we have hitherto enjoyed, and of representing with hard, systematic, and skilful work, united with pride in our moral and physical condition, the best dentists of our native country.

